# Distribution of *cfr* in *Staphylococcus* spp. and *Escherichia coli* Strains from Pig Farms in China and Characterization of a Novel *cfr*-Carrying F43:A-:B- Plasmid

**DOI:** 10.3389/fmicb.2017.00329

**Published:** 2017-02-28

**Authors:** Xiao-Qin Liu, Jing Wang, Wei Li, Li-Qing Zhao, Yan Lu, Jian-Hua Liu, Zhen-Ling Zeng

**Affiliations:** National Reference Laboratory of Veterinary Drug Residues, College of Veterinary Medicine, South China Agricultural UniversityGuangzhou, China

**Keywords:** *cfr*, *Escherichia coli*, F43:A-:B- plasmid, *Staphylococcus* spp., swine

## Abstract

The multi-resistance gene *cfr* is widely distributed among various gram-positive and gram-negative species in livestock in China. To better understand the epidemiology of *cfr* among *Staphylococcus* spp. and *E. coli* isolates, 254 *Staphylococcus* spp. and 398 *E. coli* strains collected from six swine farms in China were subjected to prevalence and genetic analysis. Forty (15.7%) *Staphylococcus* spp. isolates, including 38 *Staphylococcus sciuri* strains, one *Staphylococcus chromogenes* strain, and one *Staphylococcus lentus* strain, and two (0.5%) *E. coli* isolates were found to contain the *cfr* gene. Most of the 38 *S. sciuri* strains were clonally unrelated; however, clonal dissemination of *cfr*-positive *S. sciuri* was detected at the same farm. In eight randomly selected *cfr*-positive staphylococci, a *cfr*-harboring module (IS*21-558-cfr-*Δ*tnpB*) was detected in six *S. sciuri* isolates; *cfr* was bracketed by two copies of IS*Enfa4* or IS*256* in the remaining two *S. sciuri* isolates. In the two *E. coli* isolates, EP25 and EP28, *cfr* was flanked by two IS*26* elements in the same or opposite orientation, respectively. Complete sequence analysis of the novel F43:A-:B- plasmid pHNEP28 revealed that it contains two multi-resistance regions: *cfr* together with *floR*, *qnrS1* interspersed with IS*26*, ΔIS*CR2* and IS*Kpn19*, and *bla*_TEM-1_ together with *tet*(M) interspersed with IS*26*, IS*Apl1*, ΔTn*2*, and ΔIS*1B*. The coexistence of *cfr* with other resistance genes on a conjugative plasmid may contribute to the dissemination of these genes by co-selection. Thus, rational drug use and continued surveillance of *cfr* in swine farms are warranted.

## Introduction

The multi-resistance gene *cfr* encodes a methyltransferase that modifies position A2503 at 23S rRNA and confers resistance to six different classes of antibiotics that bind to the peptidyl transferase center (Kehrenberg et al., 2005; Giessing et al., 2009). Cfr confers resistance to phenicols, lincosamides, oxazolidinones, pleuromutilins, and streptogramin A antibiotics (the PhLOPS_A_ phenotype) and reduces susceptibility to the 16-membered macrolides josamysin and spiramycin (Long et al., 2006), which are used extensively in the treatment of infections in both humans and animals. Oxazolidinones, in particular, are regarded as the last defense against methicillin-resistant *Staphylococcus aureus* (MRSA) or vancomycin-resistant enterococci in humans (Toh et al., 2007). The *cfr* gene was first identified in a bovine *Staphylococcus sciuri* isolate (Stefan et al., 2000) and subsequently in staphylococcal isolates of human and animal origin from around the world (Shen et al., 2013). In addition, various *Enterococcus*, *Bacillus*, *Macrococcus*, *Jeotgalicoccus*, *Streptococcus suis*, *Proteus vulgaris*, and *Escherichia coli* strains have also been reported to be *cfr*-positive (Shen et al., 2013; Wang et al., 2013b). The *cfr* gene is not only widely disseminated in staphylococci from pigs in China (Shen et al., 2013), but also increasingly reported in porcine *E. coli* isolates in China. To date, a total of 16 *cfr*-positive *E. coli* isolates have been identified in the Shandong, Sichuan, Guangxi, and Guangdong provinces, China (Deng et al., 2014; Wang et al., 2012a; Zhang et al., 2014; Zhang R. et al., 2015; Zhang W.J. et al., 2015), suggesting the possibility of the transfer of *cfr* from *Staphylococcus* to *E. coli*. In this study, we identified *cfr*-positive *Staphylococcus* spp. and *E. coli* isolates from the same farm and determined the complete nucleotide sequence of a novel conjugative F43:A-:B- plasmid bearing *cfr* in an *E. coli* isolate.

## Materials and Methods

### Sampling and Bacterial Isolates

A total of 323 pig nasal swab samples and 442 fecal samples were collected in 2013 from six swine farms located in four geographically distinct provinces in China: one farm in Jilin (DBNA), two farms in Guangdong (GDEP and GDYD), one farm in Jiangxi (JXTC), and two farms in Henan (HNYM and HNYC). The usage of antibiotic agents at these farms over the past year (except farm DBNA) was supplied by their veterinarians. All animal studies were performed with the approval of the Animal Care and Use Committee of South China Agriculture University. Nasal swab samples were incubated in 7.5% Sodium Chloride Broth for 16–18 h at 37°C and then streaked onto Mannitol Salt agar plates and grown for 16–18 h at 37°C. Next, one presumptive staphylococcal isolate per sample was randomly selected for further analysis. Fecal samples were used to isolate *E. coli* as previously described (Chen et al., 2007). Species identification of all *cfr*-positive strains was determined by the ID32 STAPH system (bioMérieux, Craponne, France) and was further confirmed by sequencing the 16S rRNA using universal prokaryotic primers (Weisburg et al., 1991).

### Identification of Resistance Genes and the Genetic Environment of *cfr*

All *Staphylococcus* spp. and *E. coli* isolates were screened for the presence of *cfr* by polymerase chain reaction (PCR) as previously described (Kehrenberg and Schwarz, 2006). The *cfr*-positive staphylococci were also PCR-screened for the presence of the *fexA*, *fexB*, *mecA*, and *mecC* genes (Murakami et al., 1991; Kehrenberg and Schwarz, 2006; Liu et al., 2012; Paterson et al., 2012). Additionally, the *cfr*-positive *E. coli* strains were screened for *floR* (Chen et al., 2004).

The genetic context surrounding *cfr* was examined by PCR mapping and sequencing in eight randomly selected *cfr*-positive *Staphylococcus* spp. and two *cfr*-positive *E. coli* isolates. The primers used to determine the regions upstream and downstream of *cfr* are listed in Supplementary Table S1.

### Antimicrobial Susceptibility Testing

All *cfr-*positive *Staphylococcus* spp. and *E. coli* isolates were tested for their antimicrobial susceptibility using the agar dilution method. Antimicrobial susceptibility tests were conducted and evaluated according to the recommendations specified in CLSI documents VET01-S2 (CLSI, 2013) and M100-S25 (CLSI, 2015). Minimal inhibitory concentrations (MICs) of ≥16 mg/L were tentatively considered as criteria of florfenicol resistance (Kehrenberg and Schwarz, 2006). *S. aureus* ATCC^®^29213 and *E. coli* ATCC^®^25922 served as quality control strains.

### Multilocus Sequence Typing (MLST)

Seven housekeeping genes, including *adk, fumC, gyrB, icd, mdh, purA*, and *recA*, were used to determine the sequence types (STs) of all the *cfr*-positive *E. coli* (Lau et al., 2008). The MLST databases were used for BLAST analysis^1^.

### Pulsed-Field Gel Electrophoresis (PFGE)

Pulsed-field gel electrophoresis (PFGE) was used to determine the clonality of the *cfr*-positive *S. sciuri* isolates. Whole-cells were packed with an equal volume of 2% (wt/vol) low melting point agarose (Bio-Rad, Laboratories, Hercules, CA, USA) dissolved in EC buffer (Liu et al., 2014) and poured into plug molds. The frozen plugs were addressed according to the previously described with minor modification. The digested fragments were separated using a CHEF-DRIII system (Bio-Rad) with a clamped homogeneous electric field of 6 V/cm, using a 120° switch angle for 24 h at 14°C, with the pulse time linearly ramped from 3 to 40 s. PFGE was performed for all *cfr*-positive *E. coli* isolates using the CHEF Mapper System (Bio-Rad), according to a previously described protocol (Chen et al., 2007). Comparison of PFGE patterns was performed using the BioNumerics software (Applied Maths, Sint-Martens-Latem, Belgium) using the Dice coefficient (1.5% optimization and 1.0% tolerance); a similarity cutoff of 80% was used to identify a PFGE cluster.

## S1-Pfge, Conjugation/Transformation, and Replicon Type of *cfr*-Carrying Plasmids from *E. coli*

S1 nuclease pulsed-field gel electrophoresis (S1-PFGE) combined with Southern blotting was performed to determine the location of *cfr* in *E. coli* and the size of the plasmid according to previous protocol with minor modifications (Barton et al., 1995). Conjugation experiments were conducted using *E. coli* C600 (streptomycin resistant) as the recipient. Transconjugants were selected on MacConkey agar containing 3000 mg/L streptomycin and 10 mg/L florfenicol. Plasmid DNA was extracted from *cfr-*carrying *E. coli* strains and transformed into *E. coli* recipient strain DH5α (Takara) using the calcium chloride method when the *cfr*-carrying plasmid could not be transferred to *E. coli* C600 via conjugation. Transformants were selected on Luria-Bertani agar plates containing 10 mg/L florfenicol. The replicon types of *cfr*-carrying plasmids were determined by PCR-based replicon typing (PBRT), as previously described (Carattoli et al., 2005). Replicon sequence typing (RST) was performed to further characterize the IncFII plasmid (Villa et al., 2010).

### Sequencing of Plasmid pHNEP28

Plasmid pHNEP28 from porcine *E. coli* isolate EP28 was purified from a transconjugant using a Qiagen plasmid midi kit (Qiagen, Hilden, Germany) and 3 μg DNA was quantified by NanoDrop^TM^ 2000/2000c Spectrophotometers from Thermo Scientific. Sequence was performed by the Roche 454 GS-FLX system. Contigs were assembled with the 454 GS de novo assembler (Newbler) v2.8. Gaps between contigs were closed by PCR and sequencing. Annotation of pHNEP28 was performed using the RAST server (Aziz et al., 2008), ISfinder^2^, and BLAST^3^.

### Statistical Analysis

Statistical significance for the comparison of prevalence data was determined by the *x*^2^-test. *p-*values less than 0.05 were regarded as statistically significant.

### Nucleotide Sequence Accession Number

The nucleotide sequence of the plasmid pHNEP28 has been deposited with the GenBank nucleotide sequence database under accession number KT845955.

## Results

### Detection of *cfr* and Antimicrobial Susceptibility of *cfr*-Positive Isolates

Two-hundred and fifty four *Staphylococcus* spp. strains and 398 *E. coli* strains were recovered from the 323 nasal swab and 442 fecal samples, respectively (**Table 1**). Among them, *cfr* was detected in 40 (15.7%) *Staphylococcus* spp. and two (0.5%) *E. coli* isolates. Farm GDEP in the Guangdong province showed the highest prevalence of *cfr*-positive (37.5%) *Staphylococcus* spp. strains and it was also the only farm with *cfr*-positive *E. coli* strains (**Table 1**). The predominant *cfr*-positive *Staphylococcus* spp. species was *S. sciuri* (*n* = 38); additionally, one *S. chromogenes* isolate was obtained from farm HNYC and one *S. lentus* isolate was found at farm GDYD.

**Table 1:** Detection rates of *cfr* and *fexA* among 40 *Staphylococcus* spp. and 2 *Escherichia coli* strains from six pig farm.

	*Staphylococcus* spp.^1^							*E. coli*			Drugs used in these farms
Farms	No. of nasal swabs	Isolates	No. of *cfr* positive (%)	No. of *mecA* and *cfr* positive	No. of *mecA* positive	No. of *fexA* and *cfr* positive	No. of *mecA*, *fexA*, and *cfr positive*	No. of fecal swabs	Isolates of *E. coli*	No. of *cfr* positive (%)
DBNA	70	46	8 (17.4)	4	15	4	2	30	26	0	n.a.
GDEP	24	24	9 (37.5)	9	18	9	9	48	41	2 (4.9)	AMC,KAN,GEN,PEN,LIN,AMP, ENO,STR,FLR
GDYD	29	20	7 (35)	7	11	4	4	49	39	0	FLR
JXTC	50	47	12 (25.5)	10	31	5	5	119	105	0	CEF,TET,FLR,TIL,LIN,TYL,AMC
HNYM	50	48	0 (0)	0	20	0	0	99	94	0	GEN,PEN,ENO,CEF,TUL
HNYC	100	69	4 (5.8)	2	52	4	2	97	93	0	GEN,PEN,ENO,CEF,TUL
Total	323	254	40 (15.7)	32	147	26	22	442	398	2 (0.5)	

*AMC, amoxicillin; CEF, cefotaxime; KAN, kanamycin; GEN, gentamicin; PEN, penicillin; LIN, lincomycin; AMP, ampicillin; ENO, enrofloxacin; STR, streptomycin; TET, tetracycline; FLR, florfenicol; TIL, tilmicosin; TYL, Acetylisovaleryltylosin Tartrate; TUL, tulathromycin; n.a., not available. ^1^ 38 Staphylococcus sciuri, 1 Staphylococcus lentus and 1 Staphylococcus chromogenes.*

### Antimicrobial Resistance and Resistance Determinants

Antimicrobial susceptibility assays showed that all 40 *cfr*-positive *Staphylococcus* spp. isolates exhibited resistance to erythromycin, tetracycline, clindamycin, and trimethoprim-sulfamethoxazole. In addition, 39 (97.5%), 37 (92.5%), 36 (90%), 36 (90%), and 34 (85%) *cfr*-positive *Staphylococcus* spp. isolates demonstrated resistance to oxacillin, tiamulin, valnemulin, florfenicol, and gentamicin, respectively. Of the 40 *cfr*-positive staphylococci, 19 (47.5%) and 11 (27.5%) were resistant to ciprofloxacin and rifamycin, while only three (7.5%) isolates were linezolid-resistant (**Table 2** and Supplementary Table S2). The two *cfr*-positive *E. coli* strains also exhibited a multiresistance phenotype, namely, ampicillin, tetracycline, streptomycin, florfenicol, and trimethoprim-sulfamethoxazole resistance. Strain EP28 was also ciprofloxacin-resistant (**Table 3**).

**Table 2:** Characteristics of 40 *cfr*-carrying *Staphylococcal* spp. isolates.

				Genotype characterization		
Isolates	PFGE pattern	*Staphylococcal* species	Resistance phenotype	*mecA*	*fexA*	Genetic environment of *cfr* gene^1^
JXTC3	A1	*S. sciuri*	TIA,VAL,OX,GEN,CIP,SXT,EM,TET,CLI	+		
JXTC7	C	*S. sciuri*	FLR,TIA,VAL,OX,FOX, CIP,SXT,EM,TET,CLI	+		
JXTC8	D	*S. sciuri*	FLR, TIA,VAL,OX,FOX,GEN, CIP,SXT,EM,TET,CLI	+		
JXTC9	A2	*S. sciuri*	FLR, TIA,VAL,OX,GEN, CIP,SXT,EM,TET,CLI	+		
JXTC11	B1	*S. sciuri*	FLR, TIA,VAL,OX,GEN, RIF, CIP,SXT,EM,TET,CLI	+		
JXTC13	A3	*S. sciuri*	FLR, TIA,VAL,PEN,AMP,OX,FOX,GEN,SXT,EM, TET,CLI	+		
JXTC17	B2	*S. sciuri*	FLR, TIA,VAL,OX, FOX,GEN,CIP,SXT,EM,TET,CLI	+	+	
JXTC28	E	*S. sciuri*	FLR, TIA,VAL,AMP,OX,GEN, CIP,SXT,EM,TET,CLI		+	ΔIS*1216-aacA-aphD-*IS*256-cfr-*IS*256*
JXTC29	F	*S. sciuri*	FLR, TIA,VAL,LNZ, OX, GEN, RIF,CIP,SXT,EM, TET,CLI	+	+	
JXTC30	G	*S. sciuri*	FLR, TIA,VAL,LNZ,AMP,OX,FOX,GEN,SXT,EM, TET,CLI		+	
JXTC31	H	*S. sciuri*	FLR,TIA,VAL,OX, RIF, CIP,SXT,EM,TET,CLI	+	+	
JXTC40	I	*S. sciuri*	FLR, TIA,VAL,OX,GEN, CIP,SXT,EM,TET,CLI	+		
GDYD26	J	*S. sciuri*	FLR, TIA,VAL,OX,FOX,GEN,SXT,EM,TET,CLI	+	+	IS*21-558-cfr-*Δ*tnpB*
GDYD27	K	*S. sciuri*	FLR, TIA,OX,GEN,SXT,EM,TET,CLI	+		
GDYD28		*S. lentus*	FLR, TIA,VAL,OX,GEN,SXT,EM,TET,CLI	+	+	
GDYD35	L	*S. sciuri*	FLR, VAL, OX,FOX,SXT,EM,TET,CLI	+	+	
GDYD36	M	*S. sciuri*	FLR, TIA,VAL,OX,FOX,GEN,SXT,EM,TET,CLI	+	+	
GDYD38	N	*S. sciuri*	FLR, TIA,VAL,OX,FOX,GEN,SXT,EM,TET,CLI	+		
GDYD39	O	*S. sciuri*	FLR, TIA,VAL,OX,FOX,GEN,SXT,EM,TET,CLI	+	+	
GDEP17	P	*S. sciuri*	FLR, VAL,OX,SXT,EM,TET,CLI	+	+	
GDEP24	Q	*S. sciuri*	FLR, TIA,VAL,OX,FOX,GEN,SXT,EM,TET,CLI	+	+	IS*21-558-cfr-*Δ*tnpB*
GDEP26	R	*S. sciuri*	FLR,TIA,VAL,OX,FOX,GEN,SXT,EM,TET,CLI	+	+	
GDEP27	S	*S. sciuri*	FLR,TIA,VAL, OX,GEN, CIP,SXT,EM,TET,CLI	+	+	IS*Enfa4-cfr-* IS*Enfa4*
GDEP39	T	*S. sciuri*	FLR,TIA,VAL,OX,GEN,SXT,EM,TET,CLI	+	+	IS*21-558-cfr-*Δ*tnpB*
GDEP40	U	*S. sciuri*	FLR,TIA,VAL,OX,FOX,GEN,SXT,EM,TET,CLI	+	+	
GDEP43	V	*S. sciuri*	FLR,TIA,VAL,OX,GEN,SXT,EM,TET,CLI	+	+	IS*21-558-cfr-*Δ*tnpB*
GDEP44	W	*S. sciuri*	FLR,TIA,VAL,OX,GEN,SXT,EM,TET,CLI	+	+	
GDEP48	X	*S. sciuri*	FLR, TIA,VAL,OX,FOX,GEN,SXT,EM,TET,CLI	+	+	
HNYC8		*S. chromogenes*	FLR, TIA,VAL,TET, RIF,SXT,EM,TET,CLI		+	
HNYC31		*S. sciuri*	FLR, TIA,VAL,PEN,AMP,OX,FOX,GEN,SXT,EM, TET,CLI	+	+	
HNYC32	Y	*S. sciuri*	TIA,VAL,OX,GEN, RIF,SXT,EM,TET,CLI		+	
HNYC49	Z	*S. sciuri*	FLR,TIA,VAL,LNZ RIF,PEN,AMP,OX,FOX,GEN, CIP,SXT,EM,TET,CLI	+	+	
DBNA9-2	AA	*S. sciuri*	FLR,TIA,VAL,OX,GEN, RIF,CIP,SXT,EM,TET,CLI			
DBNA12		*S. sciuri*	FLR,TIA,OX,GEN,RIF,CIP,SXT,EM,TET,CLI	+	+	IS*21-558-cfr-*Δ*tnpB*
DBNA15		*S. sciuri*	FLR,TIA,VAL,OX,FOX,GEN,RIF,CIP,SXT,EM,TET,CLI	+	+	
DBNA16		*S. sciuri*	FLR,TIA,VAL,OX,GEN,RIF,CIP,SXT,EM,TET,CLI		+	
DBNA21		*S. sciuri*	FLR,TIA,VAL,OX,FOX,GEN,RIF,CIP,SXT,EM,TET,CLI			
DBNA22		*S. sciuri*	OX,RIF,SXT,EM,TET,CLI	+		
DBNA23		*S. sciuri*	FLR,TIA,VAL,RIF,OX,GEN,CIP,SXT,EM,TET,CLI	+	+	
DBNA47	AB	*S. sciuri*	FLR,TIA,VAL, OX,GEN,SXT,EM,TET,CLI			IS*21-558-cfr-*Δ*tnpB*

*PEN, penicillin; OX, oxacillin; FOX, cefoxitin; AMP, ampicillin; CIP, ciprofloxacin; TIA, tiamulin; SXT, trimethoprim-sulfamethoxazole; LNZ, linezolid; VAL, valnemulin; VAN, vancomycin; CLI, clindamycin; GEN, gentamycin; EM, erythromycin; RIF, rifamycin; TET, tetracycline; FLR, florfenicol; ND, not determined. ^1^ For eight randomly selected S. sciuri isolates.*

Of the 40 *cfr*-positive staphylococcal isolates, 25 strains carried *fexA*, while *fexB* was not detected. Interestingly, 32 of the 39 oxacillin-resistant staphylococci harbored *mecA*; *mecC* was not detected in the remaining seven *mecA*-negative strains. In addition, *mecA*, *fexA*, and *cfr* coexisted in 22 staphylococci (**Table 2**). The *cfr*-positive *E. coli* strain EP28 also carried *floR* (**Table 3**).

**Table 3:** Characteristics of 2 *cfr*-carrying *E. coli* isolates and their transconjugant/transformant.

Isolates^1^	Resistance Phenotype (mg/mL)									MLST	PFGE	Genotype characterization	
	AMP	CTX	AMI	TET	GEN	STR	SXT	FLR	CIP			*floR*	Genetic environment of *cfr* gene
EP28	≥32	0.125	2	128	1	256	>64	>64	4	4241	A’	+	IS*26*-*cfr*-IS*26* in the same orientation
EP25	≥32	0.125	2	128	0.5	>256	>64	>64	0.5	1602	B’		IS*26*-*cfr*-IS*26* in the opposite orientation
C600	2	0.015	2	1	0.5	>256	0.25	2	0.008				
EP28-2J	≥32	0.125	2	2	0.5	>256	0.25	>64	0.5			+	IS*26*-*cfr*-IS*26* in the same orientation
DH5α	4	0.03	1	2	0.5	4	0.5	4	0.008				
EP25-1Z	≥32	0.125	1	32	0.5	>256	32	>64	0.06				IS*26*-*cfr*-IS*26* in the opposite orientation

*AMP, ampicillin; CTX, cefotaxime; AMI, Amikacin; TET, tetracycline; GEN, gentamycin; STR, streptomycin; SXT, trimethoprim-sulfamethoxazole; FLR, florfenicol; CIP, ciprofloxacin. ^1^ EP28 and EP25 are original cfr-positive isolates; C600 is transconjugant receptor; EP28-2J is transconjugan from isolate EP28; DH5α is transformant receptor; EP25-1Z is transformant from EP25.*

### Molecular Typing

The 38 *cfr*-positive *S. sciuri* strains were analyzed by PFGE and 28 major *Sma*I patterns were observed, whereas seven strains were nontypeable by PFGE using *Sma*I (**Table 2** and Supplementary Figure S1). Although obtained from the same farm, the two *cfr*-carrying *E. coli* isolates were not related (**Table 3**). The *cfr*-positive *E. coli* strain EP25 was shown to belong to ST1602, whereas *E. coli* EP28 represented a novel ST, ST4241 (**Table 3**). Interestingly, five *cfr*-positive *S. sciuri* strains isolated from the same farm located in the Jiangxi province were grouped in two clusters (Dice similarity index was ≥80%): strains with similar PFGE patterns A1, A2, and A3, and two strains showing related PFGE patterns B1 and B2.

### Genetic Environment of *cfr*

The regions surrounding *cfr* were determined by PCR mapping and sequencing in eight randomly selected *Staphylococcus* strains. In six isolates, IS*21-558* was identified 99% identity with upstream of *cfr* and truncated transposase gene *tnpB* was found downstream of *cfr* (**Table 2**). This arrangement (IS*21-558-cfr-*Δ*tnpB*) is identical 99% identity to the corresponding region of plasmids pSS-02 (accession no. JF834910, *S. saprophyticus*, swine, China), pHNTLD18 (accession no. KF751702, *S. equorum*, pork, China), pSA737 (accession no. KC206006, *S. aureus*, patient, USA), pHK01 (accession no. KC820816, *S. cohnii*, patient, China), and pHNCR35 (accession no. KF861983, *S. simulans*, hog market worker, China) (Wang et al., 2015).

In *S. sciuri* strain GDEP27, *cfr* was bracketed by two copies of IS*Enfa4* in the same orientation, which was identical 98% identity to the corresponding region of *E. faecalis* pW9-2 (accession no. JQ911741, sewage at pig farm, China) and pHOU-*cfr* (accession no. JQ660368, patient, Thailand), *E. thailandicus* pW3 (accession no. JQ911739, sewage at pig farm, China), and p3-38 (accession no. JQ911740, pig, China).

In *S. sciuri* strain JXTC28, *cfr* is bracketed by two copies of IS*256* in the same orientation, a partial fragment of IS*1216* and the aminoglycoside resistance gene *aacA*-*aphD* were located downstream of IS*256*. This ΔIS*1216-aacA-aphD-*IS*256-cfr-*IS*256* module was 98% identity to that found on plasmid pSS-04 (accession no. KF129410) carried by porcine *S. sciuri* GN5-1 in Sichuan province, China.

In both *cfr*-positive *E. coli* strains, the *cfr* gene was flanked by two copies of IS*26* (Supplementary Figure S2). Strain EP25 had a similar genetic environment to plasmids pEC-01 (accession no. JN982327) identified in a porcine *E. coli* strain from the Shandong province, China, and pSD7 (accession no. KJ453116) found in a porcine *E. coli* isolate from the Guangdong province, China; 2 copies of IS*26* located in the same orientation, but opposite to *cfr* gene (Supplementary Figure S2B). In EP28, the 2 copies of IS*26* are in opposite orientation relative to one another, with *cfr* in the same orientation than IS*26* right-copy (Supplementary Figure S2A).

### Transformation of Plasmids Carrying *cfr* into *E. coli*

S1-PFGE and Southern blotting showed that the *cfr* gene in isolates EP25 and EP28 were located on a plasmid of approximately 95 and 105 kb, respectively. The *cfr*-carrying plasmids from isolate EP28 and EP25, designated as pHNEP28 and pHNEP25, were successfully transferred to *E. coli* C600 and DH5α, respectively. Plasmid pHNEP28 was identified as F43:A-:B-, while the replicon type of pHNEP25 could not be determined using the PBRT method. Both the transconjugant EP28-2J and the transformant EP25-1Z exhibited resistance against ampicillin, florfenicol, and elevated MICs of cefotaxime and ciprofloxacin, compared with the recipient *E. coli* strain (C600 or DH5α; **Table 3**). In addition, EP25-1Z also showed resistance to tetracycline, streptomycin, and sulfamethoxazole-trimethoprim, whereas the acquisition of pHNEP28 increased tetracycline MIC by only onefold.

### Characterization of pHNEP28 from *E. coli* Strain EP28

**FIGURE 1:**
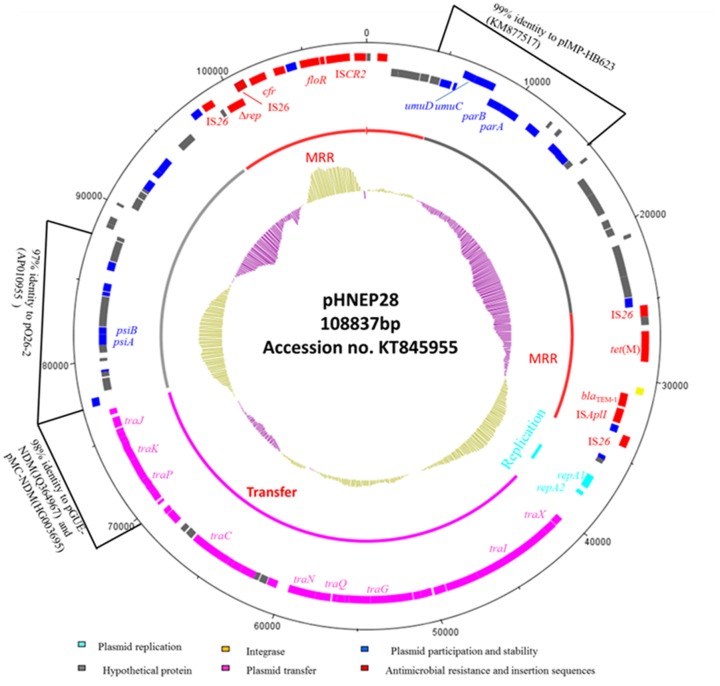
**Circular map of the IncFII:43 plasmid pHNEP28.** The most external circle represents the size of complete plasmid circle in bp. The clockwise orientation open reading frames (ORFs) are presented first and then in the counterclockwise orientation. The inner circle indicates the functional regions. The G+C plot is displayed in the inner circle, with yellow indicating >50% and purple indicating <50%.

Sequence analysis revealed that pHNEP28 is 108,837 bp in size with an average GC content of 49.85% (contigN50: 108837 bp). Plasmid pHNEP28 consisted a typical IncFII-type backbone (83,398 bp) encoding plasmid replication, horizontal transfer, maintenance and stability functions, and two multi-resistance regions (MRR) (**Figure 1**).

The replication region (position 36,852–38,269 bp), consisting of *repA2* and *repA1*, was classified as F43:A-:B:- by RST. This region shared 97% identity to two F2:A-:B- NDM-producing plasmids from *E. coli* isolates: pMC-NDM (accession no. HG003695) and pGUE-NDM (accession no. JQ364967). The transfer-leading region comprised genes related to plasmid maintenance and stability, including *parA*, *parB*, and *ssb*, as well as many putative genes. Additionally, genes involved in induced mutagenesis and DNA damage tolerance (*umuC* and *umuD*) and in the inhibition of the bacterial SOS response (*psiA* and *psiB*) were also identified. A section of the plasmid transfer-leading region (position 4,906–15,340 bp) shared 99% nucleotide identity with *Enterobacter cloacae* plasmid pIMP-HB623 (accession no. KM877517). Another section (position 77,101–88,852 bp) showed 97% identity to enteropathogenic *E. coli* plasmid p026_2 (accession no. AP010955).

The transfer region of pHNEP28 included 24 *tra* genes and *finO* and is less highly related to other IncFII plasmids, with ∼94%-97% identity to this region in *E. coli* plasmid pEB3 (accession no. CP006001) from Vietnam, which was designed to F30:A-:B:- by RST. Furthermore, the partial *tra* region of pHNEP28 (position 69475-77025 bp) showed high homology (98% nucleotide identity) with plasmids pMC-NDM and pGUE-NDM.

**FIGURE 2:**
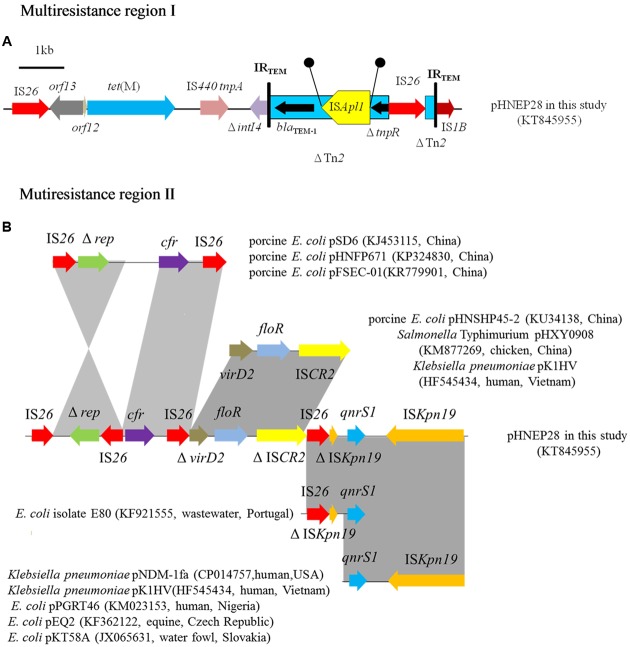
**Multiresistance regions in plasmid pHNEP28.** The arrows indicate the positions and transcription directions of the genes. Regions with >99% homology are shaded in gray. Δ indicates a truncated gene. Black circles mean direct repeats generated by the insertion of IS*Apl1*. **(A)** Multiresistance region I in plasmid pHNEP28 **(B)** Mutiresistance region II in plasmid pHNEP28 and the structural comparison with other plasmids.

The pHNEP28 contained two mosaic MRR regions (**Figure 2**). The first 9,677 bp MRR, located downstream of the replication region, contains two resistance genes (*tet*(M) and *bla*_TEM-1_) and complete or truncated insertion sequences and transposons (IS*1B*, Δ*intI4*, ΔTn2, IS*AplI*, and IS*26*). The *tet*(M) gene found in pHNEP28 shared 96–98% nucleotide identity with sequences in *S. pneumoniae* strain 11930 (accession no. FR671416), Tn*916*-like transposon Tn*6085* from *E. faecium* (accession no. HM243623), Tn*916*/Tn*1545*-like transposon from *E. faecalis* (accession no. DQ223241), and *S. aureus* strain TW20 (accession no. NG_048252); whereas it was identical to the *tet*(M) gene from porcine *E. coli* isolate CICYT-332 (accession no. KJ755873) found in Spain, although only 1802/1935 bps of CICYT-332 *tet*(M) were sequenced. While *tet*(M) demonstrated only 96% identity to that in plasmid pTCY4 (KJ772289, duck *E. coli*, China), the 1,204-bp module (IS*26*-*orf12*-*orf13*) upstream of *tet*(M) was identical to the corresponding region of pTCY4. Transposon Tn*2* carrying resistance gene *bla*_TEM-1_ was interrupted by IS*Apl1*, which generated 2-bp direct repeats (DR) (5′-CT-3′); *tnpR* of Tn*2* was truncated by IS*26*, which interrupted another Tn*2* with an opposite orientation (**Figure 2A**).

Another MRR module (14,370 bp), including the multiresistance gene *cfr*, florfenicol efflux pump gene *floR*, and quinolone resistance gene *qnrS1*, was inserted into the plasmid stability region. As shown in **Figure 2B**, the *cfr* gene was flanked by two IS*26* located in the opposite orientation, in agreement with the result obtained by PCR mapping. In addition, the 2,513-bp module (Δ*rep*-IS*26*) was identical to the corresponding region in plasmids pHNFP671 (accession no. KP324830), pSD6 (accession no. KJ453115), and pFSEC-01 (accession no. KR779901) from porcine *E. coli* in China. In plasmid pHNEP28, the Δ*rep*-IS*26* region was found in the opposite orientation with a second copy of IS*26* present in the downstream region. Furthermore, another IS*26* element is present downstream of *cfr* on plasmids pHNFP671 and pSD6, though the 2,414-bp segment containing *cfr* and IS*26* was identical to plasmid pHNEP28 in our study (**Figure 2B**).

Moreover, plasmid-mediated quinolone resistance gene *qnrS1* was flanked by two copies of IS*Kpn19* located in the opposite orientation, although the first one was interrupted by IS*26* (**Figure 2B**). The 4615-bp module (*qnrS1-*IS*Kpn19*) was present in multiple plasmids, such as *Klebsiella pneumoniae* plasmids pNDM-1fa (CP014757, human, USA) and pK1HV (HF545434, human, Vietnam), and *E. coli* plasmids pPGRT46 (KM023153, human, Nigeria), pEQ2 (KF362122, equine, Czech Republic), and pKT58A (JX065631, water fowl, Slovakia) (Dolejska et al., 2013). The IS*26*-ΔIS*Kpn19*-Δ*qnrS1* module was identical 99% to that in *E. coli* isolate E80 (KF921555, raw wastewater, Portugal), containing a disrupted *qnrS1* gene (**Figure 2B**). The 4,292-bp segment (Δ*virD2*-*floR*-ΔIS*CR2*) was identical 99% identity to the corresponding region of plasmids pHNSHP45-2 (KU34138, porcine *E. coli*, China), pHXY0908 (KM877269, chicken *Salmonella* Typhimurium, China), and pK1HV (HF545434, human *K. pneumoniae*, Vietnam); however, both *virD2* and IS*CR2* were truncated by IS*26* in plasmid pHNEP28 (**Figure 2B**).

## Discussion

In this study, *cfr* was identified in 15.7 and 0.5% of *Staphylococcus* spp. and *E. coli* isolates from pigs, respectively. The prevalence of *cfr* among *E. coli* strains was still low, which is in agreement with a previous report (Deng et al., 2014). However, to the best of our knowledge, this is the first report of *cfr* being found in *Staphylococcus* spp. and *E. coli* strains at the same farm. The presence of *cfr*-positive *E. coli* at farm GDEP may not be surprising given the high prevalence of *cfr*-positive staphylococci at that farm. The prevalence of *cfr*-positive staphylococci from farms GDEP (37.5%) and GDYD (35.5%) in the Guangdong province and from farm JXTC (25.5%) in the Jiangxi province was significantly higher than that in strains from farms HNYM (0%) and HNYC (5.8%) located in the Henan province (*p* < 0.5). According to drug usage records, florfenicol is used prophylactically or for treatment of bacterial diseases at farms GDEP, GDYD, and JXTC, but not at farms HNYM and HNYC (**Table 1**), which may account for the different detection rates. The direct selective pressure imposed by florfenicol appears to be the main driving force promoting the dissemination of *cfr* among same or different species at the same farm. This hypothesis is further supported by the presence of the florfenicol efflux gene *floR* in one *cfr*-positive *E. coli* isolate and the florfenicol efflux gene *fexA* in 25 *cfr*-carrying staphylococcal strains, which is higher than the prevalence of *fexA* among *cfr*-positive staphylococci (17/33, 51.5%) obtained from pigs in the Shandong province, China in 2011 (Wang et al., 2012b). In addition, the *cfr*-carrying isolates may also persist and be co-selected under the pressure of other agents; the results of the antimicrobial susceptibility assay reveal that the *cfr*-positive staphylococci exhibit a multiresistance phenotype, which is not limited to PhLOPS_A_.

In the present study, *S. sciuri* (*n* = 38) was the predominant species among the 40 *cfr*-positive staphylococcal strains. Previous report indicates that *S. sciuri* might be a reservoir for resistance and virulence genes both in veterinary and human medicine (Nemeghaire et al., 2014). *cfr* was first identified on plasmid pSCFS1 from a bovine *S. sciuri* in Germany (Stefan et al., 2000) and has since been detected on plasmids or the chromosomal DNA in *S. sciuri* strains from pigs and cattle in Germany (Kehrenberg and Schwarz, 2006) and from pigs, duck, chickens, and chicken meat in China (Wang et al., 2012a, 2013a; Zeng et al., 2014), indicating that *S. sciuri* is an important reservoir for the *cfr* gene, which contributes to the spread of *cfr* among animals and animal food. *Sma*I PFGE performed to further elucidate the dissemination of *cfr*-positive *S. sciuri*, revealed a variety of patterns. Most of the *cfr*-carrying *S. sciuri* isolates were clonally unrelated, indicating that the spread of *cfr* in pig farms was not due to clonal dissemination. The related PFGE patterns exhibited by a number of strains from farm JXTC, suggest that clonal dissemination of *cfr*-positive *S. sciuri* had occurred at this farm. However, the main mechanism for the dissemination of *cfr* among *S. sciuri* was probably horizontal transmission mediated by mobile genetic elements. The presence of a similar *cfr*-harboring module (IS*21-558-cfr-*Δ*tnpB*) in six randomly selected *S. sciuri*, which was identical to corresponding regions of plasmids from animals, humans, and food, may further confirm this hypothesis. In addition, *cfr* was bracketed by two copies of IS*Enfa4* or IS*256* in *S. sciuri* strain GDEP27 and JXTC28, respectively, which was highly similar to that in *Enterococcus* or *Staphylococcus* isolates, further suggesting these insertion sequences are associated with the transmission of *cfr* among same or different species from various sources.

Similarly, the two *cfr*-carrying *E. coli* showed different *Xba*I PFGE patterns and ST types. Additionally, *cfr* was flanked by two copies of IS*26* in both strains, as previously reported in other *E. coli* plasmids, whereas IS*26* in strain EP28 was in the opposite orientation. IS*26* is widespread among gram-negative bacteria and can mediate the mobility of *cfr* in gram-negative isolates (Shen et al., 2013). The *cfr*-harboring module (IS*26*-*cfr*-IS*26*) was the most prevalent genetic structure among *E. coli* strains, though *cfr* was flanked by two copies of IS*256* on plasmid pSCEC2, identified in an *E. coli* isolate (Zhang et al., 2014). IS*26* may play an important role in the dissemination of *cfr*, in accordance with previous reports (Deng et al., 2014; Zhang R. et al., 2015).

Plasmid pHNEP28 obtained in the present study from *E. coli* isolate EP28 was classified as F43:A-:B- by RST. Because *cfr* has not been detected previously in an IncFII plasmid, plasmid pHNEP28 was fully sequenced. In addition to the multiresistance gene *cfr*, pHNEP28 contains four other resistance genes including *tet*(M), *bla*_TEM-1_, *floR*, and *qnrS1*. Similar to *cfr*, *tet*(M) is widely disseminated among various gram-positive organisms and is commonly related to transposons Tn*916* and Tn*1545* (Shen et al., 2008; Ye et al., 2008; Sadowy et al., 2013). Although *tet*(M) found in pHNEP28 showed only 96–98% identity to that from gram-positive isolates and only a slightly increased (onefold) tetracycline MIC, it was identical to the *tet*(M) identified in porcine *E. coli* isolate CICYT-332 from Spain, which was classified as a new *E. coli tet*(M) allele distantly related to enterococcal *tet*(M) sequences based on phylogenetic analysis; thus, the possibility of gram-positive to gram-negative strain transfer of *tet*(M) cannot be ruled out. Additionally, the coexistence of *cfr* and other resistance genes (*tet*(M), *bla*_TEM-1_*, floR, qnrS1*) on the same plasmid not only confers resistance to multiple agents, but also may allow for co-selection of *cfr* under selective pressure imposed by other agents, thus facilitating the dissemination of *cfr*. To date, the complete DNA sequences of five *cfr*-carrying plasmids from *E. coli*, including IncA/C plasmid pSCEC2 (Zhang et al., 2014), conjugative plasmid pFSEC-01 (Zhang R. et al., 2015), IncX4 plasmid pSD11 (Sun et al., 2015), and the two similar plasmids pGXEC6 and pGXEC3, have been reported (Zhang W.J. et al., 2015). Our findings not only expand the range of plasmids that capture and spread *cfr*, but also emphasize the importance of IncFII plasmids, which have been reported to be involved in importance in the spread of many resistance genes, such as *floR*, *bla*_CTX-M_, *rmtB*, *oqxAB* (Yang et al., 2015), *bla*_CMY -2_, and *bla*_KPC_ (Del Franco et al., 2015), and *bla*_NDM_ (Fiett et al., 2014).

## Conclusion

This is the first study to demonstrate the presence of *cfr* among *Staphylococcus* spp. and *E. coli* isolates on the same farm and the first report of the complete sequence of a novel F43:A-:B- plasmid carrying *cfr*. The extensive use of florfenicol as a preventative and treatment agent in swine farms in China most likely promotes and facilitates the spread of *cfr* across species and genus boundaries, mainly via horizontal transfer mediated by mobile elements including plasmids and insertion sequences such as IS*26* and IS*256*. Thus, rational use of florfenicol and alternating antibiotic therapy are required in order to reduce *cfr* dissemination, which constitutes a potential public health risk.

## Ethics Statement

For the human cells experiments, this study was carried out in accordance with the recommendations of ethical guidelines of Chinese Academy of Sciences with written informed consent from all subjects. All subjects gave written informed consent in accordance with the Declaration of Helsinki. The protocol was approved by the Chinese Academy of Sciences.

For the zebrafish experiments, this study was carried out in accordance with the recommendations of ethical guidelines of Chinese Academy of Sciences. The protocol was approved by the ethical guidelines of Chinese Academy of Sciences.

## Author Contributions

Conceived and designed the experiments: J-HL, Z-LZ, and X-QL. Performed the experiments: X-QL, WL, L-QZ, and YL. Analyzed the data: X-QL and JW. Contributed reagents/materials/analysis tools: WL, L-QZ, and YL. Wrote the manuscript: X-QL and JW.

## Conflict of Interest Statement

The authors declare that the research was conducted in the absence of any commercial or financial relationships that could be construed as a potential conflict of interest.

## Footnotes

1 http://mlst.warwick.ac.uk/mlst/mlst/dbs/Ecoli

2 https://www-is.biotoul.fr//

3 http://blast.ncbi.nlm.nih.gov/Blast.cgi
