# Erratum: Distribution of *cfr* in *Staphylococcus* spp. and *Escherichia coli* Strains from Pig Farms in China and Characterization of a Novel *cfr*-Carrying F43:A-:B- Plasmid

**DOI:** 10.3389/fmicb.2017.00950

**Published:** 2017-05-22

**Authors:** Frontiers Production Office

**Affiliations:** Frontiers Production Office, FrontiersLausanne, Switzerland

**Keywords:** *cfr*, *Escherichia coli*, F43:A-:B- plasmid, *Staphylococcus* spp., swine

Due to a typesetting error, a funding statement which does not apply to the article was added. The following text has, therefore, been removed from the article:

This work was supported by Natural science outstanding youth fund of Shandong Province (JQ201607), Taishan Young Scholar Program of Shandong Province, AoShan Talents Program supported by Qingdao National Laboratory for Marine Science and Technology (No.2015ASTP), “100-Talent Project” of Chinese Academy of Sciences for CS.

This error does not change the scientific conclusions of the article in any way. The publisher apologizes for the mistake.

The original article has been updated.

