# Supplementary material for: Distribution of cfr in Staphylococcus spp. and Escherichia coli Strains from Pig Farms in China and Characterization of a Novel cfr-Carrying F43:A-:B- Plasmid
Source: Front Microbiol. 2017 Feb 28;8:329. doi: 10.3389/fmicb.2017.00329 (PMC5329041; doi:10.3389/fmicb.2017.00329)
Supplement: Supplementary file 1 [file Data_Sheet_1.DOCX]

**Supplementary data**

**TABLE S1 Primers used for PCR-mapping**

| Region | Primer names | Sequence(5' to 3') | Reference |
| --- | --- | --- | --- |
| IS*256*-*cfr* | IS*256*-F | GAACGCGGTTTACAAGGT | This study |
|  | *cfr*-R | ACCATATAATTGACCACAAGCAGC | 1 |
| *cfr*-IS*Enfa4* | *cfr*-F | TGAAGTATAAAGCAGGTTGGGAGTCA | 1 |
|  | IS*Enfa4*-R | GAAAATCCTTCACCAACG | This study |
| IS*21-558*-*cfr* | *istA*-F | GAGATTGTGGAAAGGCTCAAGG | 2 |
|  | *cfr*-R | ACCATATAATTGACCACAAGCAGC | 1 |
| *cfr*-Δ*tnpB* | *cfr*-F | TGAAGTATAAAGCAGGTTGGGAGTCA | 1 |
|  | *tnpB*-R | ACATGGTCCTGCAATTACTGGTA | 2 |
| IS*26*-*cfr* | IS*26*-F | GCTCTAGATCCATTCGCCAGAAAACC | 3 |
|  | IS*26*-R | GCTCTAGACTATTGGCTATTTTGATAATTACC | 3 |
| *cfr*-IS*26* | *cfr*-F | TGAAGTATAAAGCAGGTTGGGAGTCA | 1 |
|  | *26*-R | GCACGCATCACCTCAATAC | This study |

**References**

1. Wang, Y., Zhang, W., Wang, J., Wu, C., Shen, Z., Fu, X., et al., 2012. Distribution of the multidrug resistance gene *cfr* in *Staphylococcus* species isolates from swine farms in China. *Antimicrob Chemother* 56, 1485-1490.

2. Kehrenberg, C., Aarestrup, F.M., Schwarz, S., 2007. IS*21-558* insertion sequences are involved in the mobility of the multiresistance gene *cfr*. *Antimicrob Chemother 51*, 483-487.

3. Wang, Y., He, T., Schwarz, S., Zhou, D., Shen, Z., Wu, C., et al., 2012. Detection of the *staphylococcal* multiresistance gene *cfr* in *Escherichia coli* of domestic-animal origin. *J Antimicrob Chemother.* 67, 1094-1098.

**TABLE S2 Resistant percentages (%) of all staphylococcal strains from six farms**

| Farms | PEN | AMP | OX | FOX | GEN | EM | TET | FLR | CLI | VAN | TIA | VAL | RIF | SXT | CIP | LNZ |
| --- | --- | --- | --- | --- | --- | --- | --- | --- | --- | --- | --- | --- | --- | --- | --- | --- |
| NA | 54.3 | 54.3 | 89.1 | 60.9 | 84.8 | 80.4 | 100.0 | 100.0 | 97.8 | 0 | 89.1 | 78.3 | 84.8 | 43.5 | 60.9 | 4.3 |
| EP | 20.8 | 25.0 | 100.0 | 66.7 | 75.0 | 100.0 | 95.8 | 95.8 | 100.0 | 0 | 91.7 | 100.0 | 29.2 | 100.0 | 79.2 | 4.2 |
| YD | 20.0 | 35.0 | 100.0 | 55.0 | 55.0 | 100.0 | 90.0 | 100.0 | 100.0 | 0 | 80.0 | 100.0 | 15.0 | 100.0 | 70.0 | 15.0 |
| JX | 19.1 | 48.9 | 91.5 | 72.3 | 83.0 | 95.7 | 97.9 | 72.3 | 97.9 | 0 | 95.7 | 91.5 | 59.6 | 100.0 | 93.6 | 10.6 |
| YC | 76.9 | 78.5 | 95.4 | 73.8 | 81.5 | 86.2 | 98.5 | 96.9 | 95.4 | 0 | 98.5 | 93.8 | 81.5 | 66.2 | 69.2 | 4.6 |
| YM | 50.0 | 51.9 | 98.1 | 59.3 | 79.6 | 85.2 | 94.4 | 98.1 | 94.4 | 0 | 90.7 | 90.7 | 59.3 | 83.3 | 66.7 | 0.0 |
| Total | 47.2 | 55.1 | 95.7 | 66.5 | 79.9 | 89.8 | 97.6 | 94.1 | 97.6 | 0 | 93.3 | 91.7 | 63.8 | 77.6 | 73.2 | 5.5 |

PEN, penicillin; OX, oxacillin; FOX, cefoxitin; AMP, ampicillin; CIP, ciprofloxacin; TIA, tiamulin; SXT, trimethoprim-sulfamethoxazole; LNZ, linezolid; VAL, valnemulin; VAN, vancomycin; CLI, clindamycin; GEN, gentamycin; EM, erythromycin; RIF, rifamycin; TET, tetracycline; FLR, florfenicol.


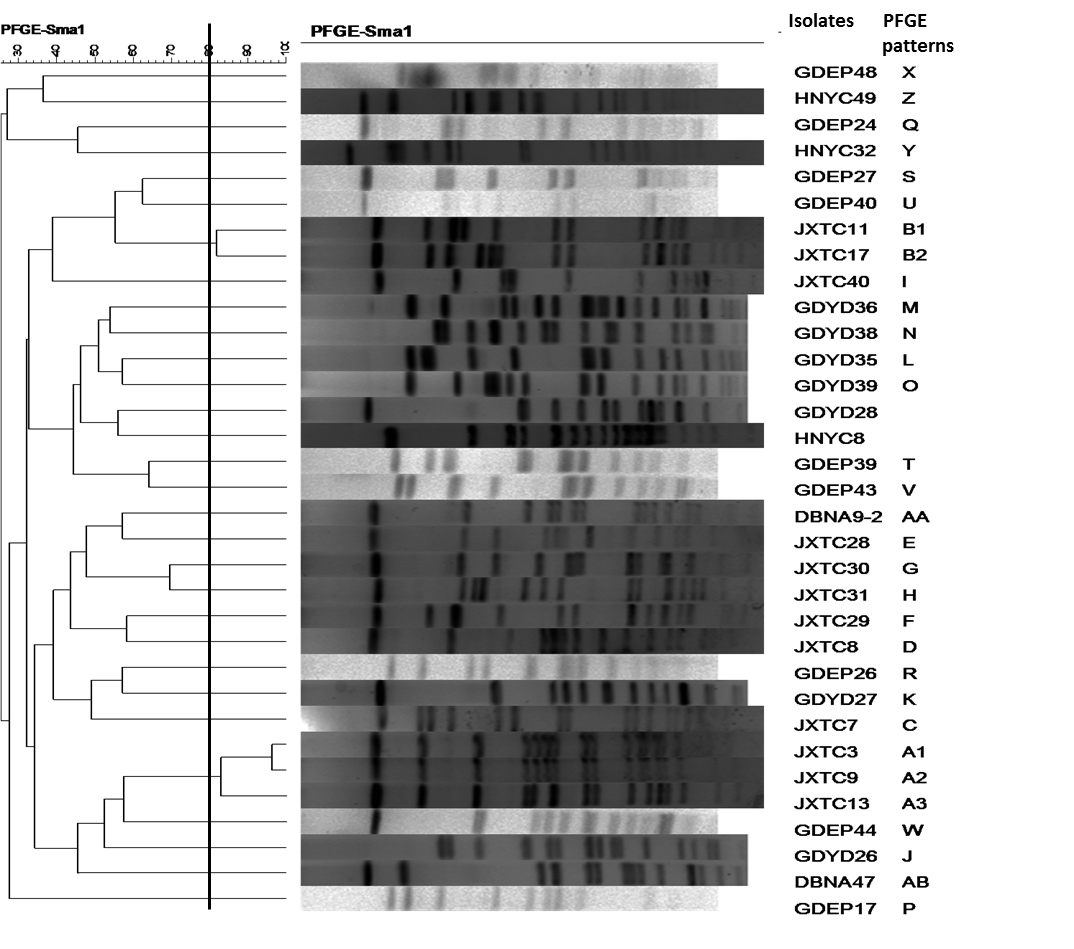


**FIGURE S1** Pulsed-field gel electrophoresis profiles of the *Sma*I-digested genomic DNA of *cfr*-positive *S. sciuri* strains

**
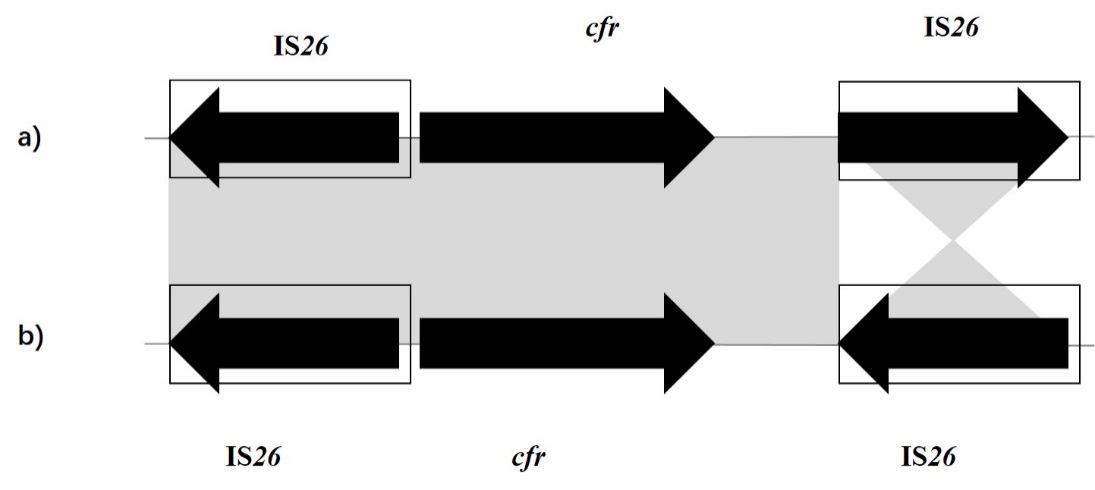
FIGURE S2** Scheme showing the genetic environment of the *E. coli* *cfr* genes identified in this study**.** The arrows indicate the position and transcriptional direction of the ORFs. Regions with over 98% homology are shaded in gray. **a**. Genetic environment of *cfr* in EP28; **b**. Genetic environment of *cfr* in EP25 and plasmid pEC-01 (accession number JN982327) from *E. coli* LYP-C-BCTb11.
